# Biological Markers of Cognitive Impairments in Combat and Contact-Sport Athletes: A Systematic Review

**DOI:** 10.3390/sports14070272

**Published:** 2026-07-01

**Authors:** José Raimundo Fernandes, Michele Andrade de Brito, Keveenrick Ferreira Costa, Felipe Inostroza Rios, Ignacio Roa-Gamboa, Naiara Ribeiro Almeida, Einstein Francisco de Camargos, Alfonso López Díaz de Durana, Bianca Miarka, Otávio de Toledo Nóbrega, Ciro José Brito

**Affiliations:** 1 Departamento de Educação Física, Universidade Federal de Juiz de Fora—Campus Governador Valadares, Governador Valadares 35010-180, Brazil; mundegv@hotmail.com (J.R.F.); felipe.inostroza.311@gmail.com (F.I.R.); ignacio.roa@estudante.ufjf.br (I.R.-G.); naiara.ribeiro@ufjf.br (N.R.A.); 2 Universidade do Vale do Rio Doce, Governador Valadares 35010-180, Brazil; michele.psi1980@gmail.com (M.A.d.B.); keveenrickf@gmail.com (K.F.C.); 3 Laboratório de Psicofisiologia e Performance em Esportes e Combates, Programa de Pós-Graduação em Educação Física, Departamento de Lutas, Universidade Federal do Rio de Janeiro, Rio de Janeiro 21941-599, Brazil; miarkasport@hotmail.com; 4 Programa de Pós-Graduação em Ciências Médicas, Universidade de Brasília, Campus Universitário Darcy Ribeiro, Brasília 70910-900, Brazil; einstein@unb.br (E.F.d.C.); otavionobrega@unb.br (O.d.T.N.); 5 Facultad de Ciencias de la Actividad Física y del Deporte, Universidad Politécnica de Madrid, 28040 Madrid, Spain; alfonso.lopez@upm.es

**Keywords:** biomarkers, cognitive dysfunction, concussion, athletes, sports

## Abstract

This systematic review investigated the direct association between biomarkers and cognitive performance in adult athletes exposed to repetitive head impacts. Searches in the APA PsycNet, PubMed, Google Scholar, CAPES, and BVSalud databases (11 April 2026) evaluated adult athletes (≥18 years) who used standardized neuropsychological tests and analyzed the association between biomarkers and cognition. Two reviewers performed selection, data extraction, and risk-of-bias assessment. Certainty of evidence was assessed using an adapted GRADE framework. A total of 10,480 records were identified. After removal of 697 duplicate records, they underwent screening and eligibility assessment, resulting in the inclusion of 10 studies. They showed low risk of bias, but sample imprecision reduced the certainty of evidence in 50% of cases. The cognitive domains assessed were memory, attention, processing speed, reaction time, and executive functions. Axonal biomarkers (70% of studies), inflammatory (40%), and synaptic biomarkers (10%). Eighty percent of the studies found a relationship between biological alterations and cognition. The most promising biomarkers associated with cognitive deficits are NfL and GFAP, but due to high methodological heterogeneity, imprecision of estimates (GRADE), and biases, the conclusions are provisional. Caution is recommended in clinical application until prospective studies with larger samples and active control groups confirm the findings.

## 1. Introduction

The growing popularity and global impact of combat sports (such as boxing, MMA, and kickboxing) and contact sports (including American football, rugby, and hockey) have highlighted concerns about the neurological health of athletes involved in these activities [1]. Although these sports are recognized for their physical and disciplinary benefits, they share an inherent risk related to frequent and repetitive exposure to head acceleration and deceleration forces [2]. These events, known as Repetitive Head Impacts (RHI), include both clinically diagnosed sport-related concussions (SRC) and numerous subconcussive impacts that do not cause immediate symptoms but occur frequently during training and competition [3]. Studies reveal that repeated subconcussive impacts can lead to microstructural brain changes and functional impairments even without an SRC diagnosis, emphasizing the cumulative neurological risks in these modalities [4].

The accumulation of RHI has been associated with a range of neurological dysfunctions, among which cognitive deficits are particularly concerning. Subtle cognitive impairments, such as slowed information processing and memory difficulties, may emerge in the acute and subacute phases [5]. Long-term exposure increases the risk of chronic cognitive impairments, such as accelerated decline in memory and executive functions, potentially culminating in neurodegenerative conditions such as chronic traumatic encephalopathy (CTE), which is strongly linked to the cumulative burden of impacts [6,7]. Epidemiological studies confirm a higher incidence of dementia among former elite contact-sport athletes compared to the general population, raising concerns about their long-term neurological health [8,9].

A major challenge lies in the fact that SRC diagnosis is essentially clinical and based on self-reported symptoms, making it inherently subjective and prone to underreporting [10]. Given this diagnostic limitation, it is crucial to identify objective, sensitive, and non-invasive methods to detect cognitive impairment at subclinical stages, before deficits become irreversible. In this context, biological markers, or biomarkers, emerge as a promising tool for sports medicine [11,12]. Proteins such as neurofilament light chain (NfL), glial fibrillary acidic protein (GFAP), Tau protein, and ubiquitin carboxy-terminal hydrolase L1 (UCH-L1) have been investigated as indicators of neuronal and glial damage [13]. Their detection in peripheral fluids (blood, saliva) provides a biological window into pathophysiological processes occurring in the brain after an impact [14,15]. The use of these biomarkers can provide quantitative data that complements clinical assessment, allowing earlier interventions and safer management of athletes’ careers and health [11].

Despite their promising potential, the scientific literature on biomarkers in athletes exposed to RHI remains fragmented and methodologically heterogeneous [12]. Previous systematic reviews have investigated the role of these markers from different perspectives: Senaratne [11] examined their utility for return-to-play decisions; Swaney [12] focused on blood biomarkers associated with secondary post-concussion outcomes; O’Connell [14] and Zetterberg and Blennow [16] discussed biological significance and clinical applicability; Meyer [17] explored relationships with broader clinical outcomes; and more recent reviews expanded the scope to alternative matrices, such as salivary biomarkers [18], or advanced toward diagnostic tools [13].

However, no previous review has specifically and systematically synthesized the direct association between biomarker levels and objective cognitive performance measured by validated neuropsychological tests in combat and contact-sport athletes. Existing reviews have predominantly addressed biomarkers in relation to symptoms, neuroimaging findings, diagnostic outcomes, or broad clinical measures, leaving the specific biomarker-cognition relationship largely unexamined [15,17]. Even with growing evidence on the temporal dynamics of these markers following brain injury, the link between biomarker concentrations and distinct cognitive domains (memory, attention, processing speed, executive functions) remains inconclusive. This gap limits the translation of biomarker research into standardized clinical protocols for the objective assessment of cognitive deficits in this population.

Filling this gap is essential for three reasons: (a) combat and contact athletes present unique patterns of impact exposure (direct blows to the head, falls, submissions), which may modulate the biomarker-cognition relationship differently from other sports; (b) early identification of subclinical cognitive impairment enables brain-protective interventions, temporary withdrawal, or career adjustments; (c) without a focused synthesis on biomarker-cognition association, it is not possible to establish evidence-based cut-off points or recommendations for longitudinal monitoring of these athletes.

Therefore, the scope of this systematic review is narrowly defined as the association between biomarkers (measured in serum, plasma, saliva, or other peripheral fluids) and objective cognitive performance (assessed by standardized neuropsychological tests) in combat and contact-sport athletes. Specifically, it seeks to: (a) synthesize evidence on the correlation between biomarker levels and neuropsychological test scores; (b) explore whether this association differs according to sport modality, career duration, or exposure frequency; (c) identify methodological gaps that hinder clinical application. Outcomes not directly related to objective cognitive performance—such as subjective symptom reports, neuroimaging findings alone, or diagnostic classifications without cognitive testing—are outside the scope of this review. By doing so, this synthesis provides a targeted, consistent foundation for evaluating the potential role of fluid biomarkers in the assessment and monitoring of cognitive impairment in this specific population.

## 2. Methods

This systematic review was conducted in accordance with the Preferred Reporting Items for Systematic Reviews and Meta-Analyses (PRISMA) 2020 statement [19] (Supplementary Materials), to ensure methodological rigor and transparency in reporting. The detailed protocol was prospectively registered in the international PROSPERO registry under the number CRD420261294872 on 28 January 2026. The review followed the registered protocol without any deviations.

### 2.1. Eligibility Criteria

The central question guiding this review was: What are the main biological markers associated with cognitive impairments in combat and contact-sport athletes? To structure the eligibility criteria, the PECOS strategy (Population, Exposure, Comparison, Outcomes, and Study type) was used [20], as detailed in Table 1.

Articles published in Portuguese, English, and Spanish were included, with no restriction on year of publication (i.e., no temporal filters were applied). Studies without empirical data, as well as those whose full text was not available after attempts to contact the authors, were excluded.

### 2.2. Information Sources and Search Strategy

The search covered the entire period available in each database, from its inception until 11 April 2026, without applying temporal filters. It was conducted in the following databases: APA PsycNet, PubMed, Google Scholar, CAPES Periodicals, and the Virtual Health Library (VHL), which integrates LILACS and IBECS.

The search strategy was designed to identify studies that investigated biomarkers and cognition in athletes of contact and combat sports. The broad search maximizes sensitivity, and specificity is achieved during the study selection phase, as recommended by the PRISMA guidelines. The more refined criteria (type of biomarker, type of cognitive assessment, analysis of association) were applied manually during the screening process. A combination of controlled vocabulary from the Health Sciences Descriptors (DeCS) and Medical Subject Headings (MeSH) was used, applied to the title, abstract, and keyword fields.

The Boolean search strategy followed the general structure: biomarkers, cognitive impairment, contact or combat sports. Synonyms within each conceptual domain were combined using the OR operator, while the three domains were connected with the AND operator. Additional resources, such as truncation (*) and quotation marks (“ ”), were used to refine the search according to each database.

No restrictions were applied regarding language, publication date, or specific database filters, in order to maximize the search. Pilot tests were conducted with full syntax in all databases, but this resulted in retrieval failures (zero results) in some of them due to technical limitations of those platforms. The strategies presented were therefore pragmatically adapted to ensure search sensitivity while maintaining the logic of the three domains (biomarkers AND cognition AND sports). The complete search strings for each database, with the appropriate adaptations, are available in the Supplementary Materials. Additionally, the reference lists of included articles and relevant prior systematic reviews were manually screened to identify further studies potentially missed in the electronic search.

### 2.3. Study Selection Process

The selection process initially took place through references retrieved from the databases, which were exported in RefMan formats and imported for analysis into the Rayyan web application (Qatar Computing Research Institute, Doha, Qatar; https://rayyan.ai; accessed on 11 April 2026) for screening and management of records [21]. The records identified in the searches were exported to the systematic review management software Rayyan for manual duplicate removal.

Next, the selection process was conducted independently and blindly by two reviewers (JRF and MAB). In the title and abstract screening, both reviewers independently assessed the potential relevance of all unique records against the eligibility criteria. Decisions regarding inclusion, exclusion, or uncertainty were recorded individually by each reviewer before any communication between them, and disagreements were resolved by a third reviewer (CJB). Inter-rater agreement for this phase was calculated using Cohen’s kappa coefficient (0.87).

Pre-selected studies advanced to the second phase (full-text screening), in which articles were retrieved and evaluated in depth, again independently, to confirm final eligibility. Inclusion or exclusion decisions in this phase were also recorded individually prior to joint discussion. Inter-rater agreement for this phase was assessed using Cohen’s kappa coefficient, which was 0.90. These results indicate excellent agreement between reviewers. Consensus was ensured by resolving all disagreements through discussion, with the intervention of a third reviewer (CJB) in cases of impasse.

**Figure 1:**
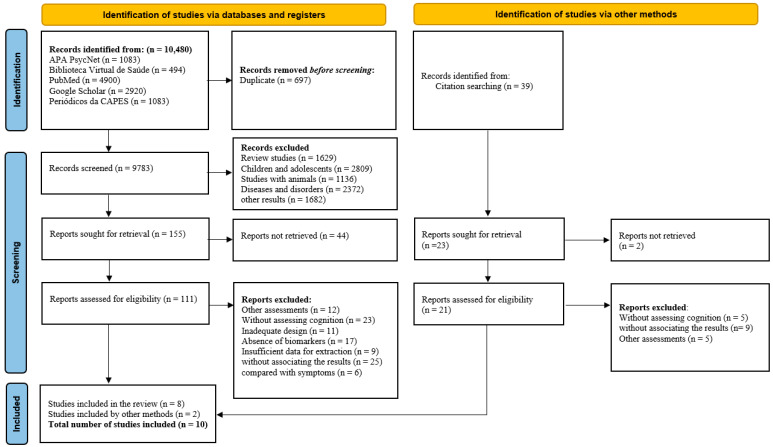
Flow diagram for new systematic reviews, which included searches of databases, registries, and other sources.

For each eligible study after full-text reading, qualitative data were extracted using a standardized form in an Excel spreadsheet. For each eligible study, the following information was extracted: (a) General characteristics: authors, year of publication, study design, sample size per group, sex distribution, age, sport modality, type of exposure (sport-related concussion or repetitive head impacts), exposure time (Career length in years/Number of fights or seasons and number of reported concussions); (b) Cognitive assessment (neuropsychological tests used); (c) Biomarkers assessed (type of biomarker); (d) Association between biomarker and cognition (direction of the association); (e) Time points (baseline, post, late follow-up). The detailed selection flow, including the number of studies excluded at each stage, is presented in the PRISMA Flow Diagram (Figure 1).

### 2.4. Risk-of-Bias Assessment

The methodological quality of the included studies was assessed using the Critical Appraisal Skills Program (CASP) Cohort Study Checklist (available at: https://casp-uk.net/casp-tools-checklists/; accessed on 11 April 2026). This tool was chosen because it is well-established and validated for observational studies with longitudinal follow-up. For the included cross-sectional studies, the same checklist was applied, but items related to follow-up (6a and 6b) were considered not applicable and were excluded from the denominator for the total score calculation (i.e., for cross-sectional studies, the maximum number of applicable items was 10; for cohort studies, 12). This decision was made a priori and is explicitly reported.

The CASP checklist comprises 12 items distributed across three sections: (A) validity of results (items 1–6b), (B) presentation of results (items 7–9), and (C) applicability (items 10–12). Table 2 presents the complete list of items with their respective wording. Each item was rated as: Yes (Y): the criterion was fully met; No (N): the criterion was not met; Cannot be determined (CD): the information provided was insufficient to make a judgment.

For the purpose of the classification criteria, internal validity items were considered to be items 1, 2, 3, 4, 5a, and 5b (a total of six items), as recommended by CASP for the proper assessment of risk of bias. Items 6a and 6b (follow-up) and items in sections B and C (presentation and applicability) were treated as complementary items.

Items 1, 2, 3, 4, 5a, 5b were defined as critical (and for cohort studies, additionally items 6a, 6b). For cross-sectional studies, items 6a and 6b were not required because they did not apply.

Based on the number of “Yes” responses (considering only applicable items) and the presence of critical flaws, studies were categorized into three levels of risk of bias:

(a) Low risk of bias (high methodological quality)—The study received ≥8 “Yes” responses out of the applicable items. Furthermore, all critical items (1, 2, 3, 4, 5a, 5b, and, for cohorts, 6a, 6b) must be rated as “Yes”. No more than one “No” response was allowed among the six internal validity items. “Cannot be determined” responses were not counted as “Yes”, but they did not automatically downgrade the study unless they reached the threshold described below.
(b) Moderate risk of bias (reasonable methodological quality)—The study received 5–7 “Yes” responses out of the applicable items OR met all critical items but had a relevant limitation in another domain (e.g., loss to follow-up >20% without sensitivity analysis; lack of adjustment for important confounders). Up to two “No” or “Cannot be determined” ratings among the six internal validity items were accepted.
(c) High risk of bias (low methodological quality)—The study received <5 “Yes” responses out of the applicable items OR failed to meet at least one critical item (1, 2, 3, 4, 5a, 5b, and, for cohorts, 6a, 6b) OR had three or more “No” responses among the six internal validity items.

Studies with four or more items rated as CD (considering all applicable items) were considered to have insufficient transparency and were downgraded by one level (e.g., from low to moderate, or moderate to high), unless additional information was obtained from the authors.

Two reviewers (JRF and MAB) independently and blindly assessed each study. Prior to the formal assessment, a pilot test was conducted on three randomly selected studies to calibrate item interpretation and ensure consistency. Disagreements between reviewers were resolved by consensus; if no consensus was reached, a third reviewer (CJB) made the final decision. The final risk-of-bias classification for each study is summarized in Table 2.

### 2.5. Data Synthesis and Descriptive Certainty Assessment Adapted from Grading of Recommendations, Assessment, Development and Evaluations (GRADE)

Given the substantial heterogeneity in study designs, biomarker types, cognitive outcomes, and sport modalities, a conventional meta-analysis was not feasible. Likewise, a full GRADE assessment, which requires pooling evidence across multiple studies for each specific outcome, could not be performed because most biomarker–outcome pairs were investigated by only one or two studies with inconsistent methodologies.

Therefore, we adopted a descriptive, study-level approach adapted from GRADE domains to avoid overinterpretation. This approach does not constitute a formal GRADE certainty assessment but rather a systematic, transparent judgment of the methodological strengths and limitations of each individual study. The findings are presented in Table 3 as a descriptive certainty rating per study, using the same five GRADE domains (risk of bias, inconsistency, indirectness, imprecision, and publication bias). Readers should interpret the “Certainty” column in Table 3 as an exploratory, study-level indicator of confidence in that particular study’s findings, not as a pooled GRADE certainty for a body of evidence across studies.

The operational definitions and downgrade criteria for each domain were defined a priori and are described below, consistent with the legend of Table 3:

(a) Risk of bias: Methodological quality was previously assessed using the CASP checklist for cohort studies. For descriptive purposes, the CASP classification was mapped as follows: “High” CASP quality = low risk of bias (good quality); “Moderate” CASP quality = moderate risk of bias; “Low” CASP quality = high risk of bias. For the adapted GRADE domain, risk of bias was considered “not critical” (no downgrade) when the study was classified as high CASP quality (low risk of bias).
(b) Publication bias: Formal assessment of publication bias using funnel plots or Egger’s test was not feasible due to the small number of studies per biomarker (a maximum of 15 eligible comparisons across different outcomes). Therefore, this domain was not downgraded for any study. However, publication bias is acknowledged as a potential limitation in Section 4.
(c) Inconsistency: Because each row in Table 3 corresponds to a single study reporting consistent results for a given biomarker–outcome pair (or a set of internally consistent findings), inconsistency across multiple effect estimates was not applicable. Hence, the “Inconsistency” column is marked N/A for all studies. For future syntheses pooling multiple studies per outcome, this domain should be reassessed.
(d) Indirectness: Indirectness was evaluated regarding population, exposure, outcome, and comparator. The domain was rated as “Direct” when the study directly assessed athletes from contact or combat sports (boxing, MMA, American football, rugby, soccer) and used objective cognitive tests (e.g., standardized neuropsychological batteries) or validated neuroimaging metrics. Indirectness would be rated as “Indirect” (with downgrade) if surrogate outcomes were used without clear correlation to clinical endpoints or if biomarker detection windows were biologically implausible. All studies in Table 3 were rated as “Direct” because only studies directly correlating biomarkers with cognitive or structural outcomes were included (see eligibility criteria).
(e) Imprecision: Imprecision was assessed based on sample size and reporting of confidence intervals (CIs), using predefined thresholds (see Table 3 legend): (1) Not critical (no downgrade): n ≥ 100 per group, or CIs consistently reported; (2) Moderate (downgrade by one level): n = 51–99, or borderline precision; (3) Serious (downgrade by one level, or two levels if extreme): n ≤ 50 per group, or sample size not reported, or no CIs provided. This domain was applied at the study level as a descriptive indicator of precision.
(f) Integration of domains and final descriptive rating: For each study, the five domains were combined to generate a descriptive certainty rating (High, Moderate, Low, or Very Low) following the general GRADE logic (starting from “High” for well-conducted observational studies and downgrading for serious limitations). Table 3 presents the domain ratings and the final descriptive certainty for each individual study. The observations column provides a brief justification for the rating.

Important caveat for interpretation: This study-level descriptive adaptation of GRADE should not be confused with a standard GRADE assessment, which synthesizes evidence across multiple studies for a single outcome. For outcomes where more than one study was available (e.g., NfL and cognitive performance), we did not pool the ratings. Instead, we present the individual study ratings descriptively. A formal GRADE certainty assessment for each biomarker–outcome pair would require a larger, more homogeneous body of evidence and is recommended for future updates of this review.

## 3. Results

The initial search in electronic databases yielded 10,480 records, and an additional 39 records were identified through other sources. After removing 697 duplicates, 9783 records proceeded to title and abstract screening. At this stage, 9628 records were excluded, with reasons including review studies (n = 1629), studies involving children and adolescents (n = 2809), animal studies (n = 1136), studies focused on diseases or disorders unrelated to the objective (n = 2372), and other outcomes (n = 1682).

Following the screening, 155 reports from database searches were sought for retrieval, of which 44 could not be obtained, leaving 111 reports assessed for eligibility. From other sources, 39 reports were identified; after excluding 2 that were not retrieved, 23 reports were assessed for eligibility.

During full-text eligibility assessment, 111 reports from database sources were excluded for the following reasons: other assessments (n = 12), absence of biomarkers (n = 17), lack of cognitive evaluation (n = 23), inadequate study design (n = 11), insufficient data (n = 9), association between biomarkers and cognition (n = 25) and compared with symptoms (n = 6). From other sources, 21 reports were excluded due to no association between biomarkers and cognition (n = 5), other outcomes (n = 9), and other assessments (n = 5).

At the conclusion of the selection process, 8 studies from database searches met all eligibility criteria and were included in the systematic review, together with 2 additional reports identified through other methods, resulting in a total of 10 included studies.

Agreement between reviewers was assessed separately for each screening phase using Cohen’s kappa coefficient. During title and abstract screening, the kappa value was 0.89, indicating substantial agreement. For full-text screening, the kappa value increased to 0.91. Overall, these results demonstrate an almost perfect level of agreement between reviewers in these stages of the selection process (*p* < 0.001).

Regarding the CASP checklist, all evaluated studies (n = 10) were classified as high quality, indicating overall methodological adequacy. Of these, 30% (cross-sectional studies), items 6a (complete follow-up) and 6b (sufficiently long follow-up) were not applicable and were not counted. Regarding item 8 (precision of results), 50% of the studies received an “S”, while 50% were classified as “U” (uncertain). No study received a score of “N” (no) for any item. It is concluded that the studies generally present methodological robustness.

In the Grade analysis, all 100% of the studies presented a low risk of bias, starting from a high initial certainty. In the domain of imprecision, 50% of the studies were classified as “Non-critical”, 20% as “Moderate”, and 30% as “Serious”. The domains of publication bias and inconsistency (not formally assessed) did not result in downgrading. The indirect evidence domain was classified as “Direct” for all studies.

Studies with very low certainty were justified solely by serious imprecision (small sample size). Studies with high certainty had adequate samples (n ≥ 100) and adequate follow-up when applicable. In summary, despite the low risk of methodological bias in all studies, sample imprecision was the determining factor for downgrading the certainty of evidence in 50% of the studies (30% very low certainty + 20% moderate certainty).

Table 4 summarizes the main characteristics of the 10 eligible studies. Regarding study design, the majority adopted a cohort design (70%), while 30% were cross-sectional. The sex distribution of participants was predominantly male (90.5%) and female (9.5%). Concerning sample size, 50% of the studies had small samples (n ≤ 50) and 50% had large samples (n ≥ 100). Of the total, 90% included a control group (unexposed or minimally exposed) and 10% did not. Regarding sport modalities, 56.5% involved contact sports and 43.5% involved combat sports. Regarding exposure, 60% of the studies showed that greater exposure to repetitive head impacts was associated with worse cognitive performance, and 40% showed that greater exposure was associated with no cognitive impairment. Chronic exposure (throughout the career or after retirement, with ≥ 2 years of inactivity) occurred in 70% of studies; short-term exposure (one season or initial subacute/chronic phase: 1–6 days to ≥ 3 weeks) occurred in 20%; one study did not report the follow-up time. Participants’ ages ranged from 20 to 58 years. These characteristics explain the substantial methodological heterogeneity that precluded meta-analysis and conventional GRADE synthesis.

Table 5 presents the studies that investigated the relationship between biomarkers and cognitive performance in athletes exposed to repetitive head impacts. The most frequently used instruments were the CNS Vital Signs, Trail Making Test, Digit Span, RBANS, CogState Battery, RAVLT, and ImPACT. A predominance of functions related to memory, attention, processing speed, reaction time, and executive functions was observed. Regarding the biomarkers investigated, there was a predominance of axonal markers, present in 70% of the studies; NfL appeared in 50%, GFAP in 40%, tau and its phosphorylated forms in 40%, and inflammatory biomarkers in 40% of the studies, while synaptic markers were investigated in only 10% of the studies.

Concerning the association between biomarkers and cognition, 80% of the studies identified some relationship between biological alterations and cognitive or neurobehavioral performance. In contrast, 20% found no significant associations between biomarkers and cognition. Regarding the timing of assessments, methodological variability was observed across studies. Acute post-injury evaluations were performed in 30% of the investigations, with collections ranging from minutes to a few days after the impact. Another 30% of the studies adopted longitudinal designs with annual follow-ups ranging from two to five years. Studies monitoring multiple post-injury time points accounted for 20% of the sample, while the remaining studies performed baseline assessments associated with extended follow-up periods or cross-sectional comparisons.

## 4. Discussion

This systematic review synthesized studies investigating blood biomarkers associated with cognitive impairment in combat and contact-sport athletes. Twelve eligible studies were identified from 10,480 initial records, corresponding to a yield of 0.09%. This low number reflects the specificity of our eligibility criteria, which includes adult athletes, exposure to repetitive head impacts, objective cognitive assessment, and a direct correlation between biomarkers and cognition.

Our findings are consistent with systematic reviews in the field of SRC, especially those related to guidelines. Studies by Patricios [32] and McCrory [33] highlight the fragmentation of the literature and methodological diversity as central challenges for evidence synthesis in this area, emphasizing the need for separate analyses due to physiological differences in responses to traumatic brain injury. These numbers evidence a persistent gap: despite a large volume of publications, well-designed primary studies that answer specific questions about blood biomarkers and cognition in athletes exposed to repetitive head impacts remain scarce.

The broader literature on biomarkers in mTBI shows that systematic reviews frequently encounter a limited base of eligible studies, even with less restrictive criteria. One systematic review on salivary biomarkers in mTBI identified only 7 studies after screening 524 articles, reporting a similar proportion of retained studies relative to the total records assessed [34]. Likewise, a systematic review on salivary and urinary biomarkers for mTBI included 29 studies from 27 populations, showing that even with broader criteria, the number of high-quality primary studies remains limited [35]. Another systematic review on blood biomarkers for risk stratification of mTBI in the emergency department included only six studies after applying eligibility criteria to 1824 studies, reinforcing that the scarcity of well-designed studies is a cross-cutting challenge in the field [36].

In our review, the vast majority of identified records were excluded because: 16.6% were review studies; 28.7% involved children and adolescents; 11.6% were animal studies; 24.0% involved diseases; and 17.0% had outcomes that did not associate cognition with blood biomarkers, leaving 2,1% of articles for retrieval.

### 4.1. Methodological Quality and Risk of Bias

The assessment of methodological quality and the certainty of evidence constitutes a fundamental pillar for the translation of scientific knowledge into clinical and public health recommendations [37,38,39]. The findings of the present study reveal a crucial dichotomy. Although the individual methodological robustness of the included studies was consistently high (as assessed by the CASP checklist), the global certainty of the evidence was frequently penalized due to issues of sample imprecision [40,41].

All evaluated studies (n = 10) were classified as high quality by the CASP checklist, indicating rigorous compliance with criteria for internal validity and reporting clarity. The application of CASP in systematic reviews is widely recognized as an effective tool for identifying methodological rigor, especially in qualitative and observational studies [37]. The absence of “N” (no) scores for any CASP item reinforces the careful selection of primary studies in this review, suggesting that selection and measurement biases were minimized at the source.

The non-applicability of items 6a (complete follow-up) and 6b (sufficient follow-up time) in 30% of the studies is justified by the cross-sectional design of these works. The literature corroborates that CASP’s flexibility allows for its integration with GRADE, provided that the intrinsic limitations of the study design are duly considered [42].

Despite the low probability of methodological bias (100% of studies with low risk), the certainty of evidence was downgraded in 50% of cases. This phenomenon is predominantly explained by the domain of imprecision. In the GRADE system, imprecision is evaluated not only by statistical significance but also by the width of confidence intervals (CI) and compliance with the Optimal Information Size (OIS) [43].

Our results corroborate the updated guidelines by Schünemann [44] and Zeng [45], which emphasize that the certainty of evidence should be downgraded when CIs are wide enough to include both clinically important benefits and harms, or when the sample size is insufficient to ensure the stability of estimates. In our sample, studies with “very low certainty” were justified exclusively by serious imprecision (small sample size), while studies with “high certainty” presented adequate samples (n ≥ 100), which is a common indicator of OIS in observational study contexts [43].

The literature indicates that imprecision is one of the most frequent reasons for downgrading evidence in Cochrane systematic reviews, affecting approximately 25–26% of outcomes, with small sample sizes and wide CIs being the most common reasons [46]. In the context of cross-sectional and observational studies in public health, the reduced sample size limits the statistical power for generalizations, justifying the downgrade in certainty even in methodologically robust studies [37].

The distinction between “individual study quality” and “certainty of the body of evidence” is crucial. A study may be conducted with technical excellence (high CASP), but if its sample size is small, it contributes “fragile” evidence for decision-making (low GRADE) [47,48].

This finding underscores the need for primary studies with more robust a priori sample size calculations. The observed methodological robustness (low risk of bias in all studies) is a positive point, but imprecision acts as the main obstacle to obtaining high-certainty evidence. Therefore, it is recommended that future investigations prioritize increasing sample size to strengthen the evidence base on the topic, especially in observational studies where the initial certainty of evidence is often classified as low [37,43,46,47,49].

### 4.2. Characteristics of Included Studies

Regarding the characteristics of the eligible studies, the findings present both consistencies and substantial methodological limitations. Concerning study design, the predominance of cohorts is theoretically supported as the most appropriate approach for investigating the association between chronic exposures, such as RHI, which aligns with the recommendations of the Amsterdam consensus statements [32,50] that prioritize long-term effects in athlete safety research.

However, the presence of 30% cross-sectional studies precludes the possibility of robust causal inference, as such designs are particularly vulnerable to survival and memory biases, especially in samples of older former athletes [51]. Regarding sex distribution, the predominantly male sample reflects the historical reality of participation in contact and combat sports but precludes the generalization of findings to female athletes, since current evidence shows that women have a higher risk of concussion and persistent symptoms following similar exposure [52,53]. This underrepresentation violates the recommendations of the Berlin consensus statement [33] and constitutes a critical gap.

Sample size, equally divided between small and large samples, also generates controversy. Studies with small sample sizes lack statistical power to detect modest effects, typical of subtle neurocognitive deficits in chronic traumatic encephalopathy (CTE), and tend to overestimate associations or produce false negatives [54]. On the other hand, the presence of a control group in 90% of studies is a positive and theoretically grounded point, as it allows distinguishing the effects of exposure from normal aging. However, the absence of active controls and the lack of quantification of prior exposure during childhood/adolescence introduce significant residual confounding bias, undermining the internal validity of most studies [55].

Regarding sports modalities, the distribution between contact and combat sports is heterogeneous from a biomechanical point of view: combat sports generate high-magnitude impacts but low frequency, whereas American football and hockey produce thousands of low-magnitude subconcussive impacts per season [56]. Aggregating these modalities without stratification is theoretically unsustainable and precludes any direct comparability between studies [57,58].

The pattern “the greater the RHI exposure, the lower the cognitive performance” holds true in most circumstances studied, but not all. The presence of 40% of studies with null effects reflects specific methodological limitations (acute exposure, low power, distinct modalities) and not a robust refutation of the hypothesis. Therefore, the direction of the effect is predominantly negative, though not absolute, which justifies recommendations for cognitive monitoring of athletes with chronic exposure while also highlighting the need for method standardization in future research [50,51].

Exposure time also presents critical heterogeneity: most studies assessed chronic exposure, others assessed short-term exposure, and only one study did not report the period. Although the analysis of late effects justifies chronic studies and the analysis of acute effects justifies short-term studies [9,33,59], mixing these temporal windows precludes any meta-analysis. Furthermore, in chronic studies with former athletes aged 50–60 years, the absence of baseline cognitive measures pre-exposure confounds the effect of normal brain aging with the effect of impacts, and survival bias is not controlled, undermining universal causal inference. The wide age range (20 to 58 years) allows studying different stages, but the lack of age standardization across studies that assess the same outcome is problematic [60].

In summary, the findings confirm the high heterogeneity of the field of head impacts in sports, but also preclude the possibility of consolidated conclusions, exposing important biases: female underrepresentation, absence of active controls, lack of standardization of cumulative exposure dose, and confounding by aging and survivor selection. It is recommended that future studies adopt prospective designs, sex-balanced samples, active controls, and baseline pre-exposure measurements, according to the 2021 Concussion in Sport Group consensus [61].

### 4.3. Cognitive Assessment, Biomarkers, and Results

Cognitive findings reveal that processing speed, reaction time, and sustained attention are among the cognitive domains most vulnerable to repetitive head impacts (RHI), often exhibiting subtle impairments before deficits become evident in broader cognitive functions. These domains depend on the integrity of large-scale white matter networks, which are particularly susceptible to diffuse axonal injury resulting from repetitive biomechanical forces [2,62,63]. In contact-sport athletes, significant reductions in reaction time have been observed following competitive seasons, suggesting that cumulative subconcussive exposure may negatively affect neural transmission efficiency even in the absence of clinically diagnosed concussion [64]. Interestingly, although verbal memory and executive functions are frequently assessed, deficits tend to be more consistently detected in measures requiring psychomotor speed, attentional control, and cognitive flexibility [9,65,66,67]. This pattern is consistent with evidence indicating that white matter disruption preferentially affects frontostriatal and frontoparietal circuits involved in rapid information processing [68].

A notable finding is that average cognitive performance may remain within normal limits in groups of former professional athletes despite the presence of altered neurobiological markers [15,16,69]. This phenomenon may be explained by the concept of cognitive reserve, whereby lifelong intellectual enrichment, athletic expertise, and compensatory neural recruitment temporarily preserve cognitive functioning despite ongoing neuropathological changes [62,63]. Neuroimaging studies further suggest that functional compensation may mask underlying structural damage until neurodegenerative processes reach a critical threshold, at which point cognitive decline becomes clinically apparent [62].

Biomarker analyses provide important insights into the pathophysiological mechanisms underlying RHI. Among currently available biomarkers, neurofilament light chain (NfL) and glial fibrillary acidic protein (GFAP) appear to be the most sensitive indicators of ongoing neural injury. NfL is a structural component of axons released into extracellular fluids following axonal damage and has been consistently associated with cumulative head impact exposure and reduced psychomotor and processing speed performance [15,16]. Elevated GFAP concentrations, reflecting astroglial activation and injury, have been associated with broader impairments in memory, reaction time, and global cognitive performance, particularly among retired boxers and former professional athletes [13,70].

Although total tau and phosphorylated tau isoforms (p-tau181 and p-tau231) have been identified at abnormal concentrations in former athletes and are strongly implicated in the neuropathology of chronic traumatic encephalopathy (CTE), their direct association with contemporaneous cognitive performance appears less consistent than that observed for axonal injury markers [51,71]. This observation supports the hypothesis that tau-related alterations may reflect the accumulation of chronic neurodegenerative processes, whereas NfL and GFAP are more sensitive indicators of acute and subacute tissue injury [51,72]. Furthermore, pathological tau aggregation may precede measurable cognitive symptoms by several years, contributing to the discrepancy between biomarker abnormalities and clinical presentation [73].

The role of neuroinflammation has also gained increasing attention. Elevated concentrations of inflammatory cytokines, including IL-6 and IL-8, have been associated with both cognitive dysfunction and neurobehavioral disturbances such as impulsivity, emotional dysregulation, and mood symptoms [74,75]. Chronic neuroinflammatory activation is believed to contribute to progressive neuronal dysfunction through mechanisms involving microglial activation, oxidative stress, and blood–brain barrier disruption [72,76,77]. Additionally, alterations in catecholaminergic pathways, including reductions in norepinephrine and dopaminergic metabolites, suggest that dysfunction of neuromodulatory systems may precede or accompany cognitive decline in former athletes exposed to repetitive head trauma [1,9,66]. Such alterations may contribute not only to executive dysfunction but also to emotional and autonomic disturbances frequently reported in this population [78].

Emerging evidence also supports the potential utility of circulating microRNAs (miRNAs) as minimally invasive biomarkers of brain injury. Specific salivary and serum miRNA profiles have demonstrated promising diagnostic and prognostic value for traumatic brain injury and have been associated with cognitive performance, postural control, and symptom burden [79]. Given their accessibility and sensitivity to molecular changes occurring early after injury, miRNAs may represent an important advance in the early detection and monitoring of RHI-related neurological alterations [79,80].

Taken together, these findings reinforce the notion that monitoring athletes chronically exposed to RHI should extend beyond traditional cognitive testing. A multimodal approach integrating axonal biomarkers such as NfL and GFAP, neurobehavioral assessments capable of detecting emotional dysregulation, and sensitive measures of processing speed and reaction time may provide a more comprehensive framework for identifying at-risk athletes before pathology progresses to irreversible clinical conditions, including chronic traumatic encephalopathy and dementia [55,81].

## 5. Conclusions

NfL and GFAP biomarkers are the most promising candidates associated with cognitive deficits (processing speed, memory, and reaction time) in athletes exposed to repetitive head impacts. However, due to high methodological heterogeneity, imprecision of estimates (GRADE), and the presence of significant biases, these conclusions are provisional and require confirmation through prospective studies with larger samples, active control groups, and standardization of exposure. Caution is recommended in the clinical or translational application of these findings until higher-certainty evidence becomes available.

## Figures and Tables

**Table 1:** Description of the PECOS Strategy.

Category	Inclusion Criteria	Exclusion Criteria
Population (P)	Adult athletes (≥18 years), active or retired, from combat or contact sports with direct exposure to head impacts.	Individuals with pre-existing neurological pathologies not related to sport.
Exposure (E)	Documented history of participation in combat or contact sports, including repeated subconcussive impacts or clinically diagnosed sport-related concussion (SRC).	Studies focused exclusively on traumatic brain injury (TBI) from external causes not related to sport.
Comparator (C)	Observational studies that include at least one comparison group: athletes with vs. without cognitive impairment; combat/contact-sport athletes vs. non-contact-sport athletes, or healthy individuals. Studies without a comparator group that objectively assess the association between biomarkers and cognition.	Studies that do not assess cognition or biomarkers objectively.
Outcomes (O) (a) Biomarkers	Measurement of peripheral biological biomarkers (blood, saliva, cerebrospinal fluid) or molecular markers associated with neuronal damage, neuroinflammation, or synaptic plasticity.	Studies based exclusively on imaging biomarkers (e.g., MRI, EEG) without biological fluid collection.
(b) Cognition	Objective assessment of cognitive performance using validated neuropsychological tests.	Studies reporting only subjective symptoms, self-reported cognitive complaints, or non-standardized screenings.
(c) Association	Studies that explicitly analyze the correlation between biomarkers and cognition.	Studies that assessed only biomarkers (without corresponding cognitive measures) or only cognition (without biomarkers).
Study Design (S)	Observational studies: cross-sectional, cohort (prospective or retrospective), and longitudinal studies.	Gray literature without peer review, reviews, editorials, letters, opinions, or conference abstracts.

**Table 2:** Evaluation of studies using the CASP Checklist: For Cohort Studies.

Study	1	2	3	4	5a	5b	6a	6b	7	8	9	10	11	12	Classification
[22]	Y	Y	Y	Y	Y	Y	Y	Y	Y	U	Y	Y	Y	Y	High
[23]	Y	Y	Y	Y	Y	Y	Y	Y	Y	Y	Y	Y	Y	Y	High
[24]	Y	Y	Y	Y	Y	Y	Y	Y	Y	Y	Y	Y	Y	Y	High
[25]	Y	Y	Y	Y	Y	Y	-	-	Y	U	Y	Y	Y	Y	High
[26]	Y	Y	Y	Y	Y	Y	-	-	Y	U	Y	Y	Y	Y	High
[27]	Y	Y	Y	Y	Y	Y	Y	Y	Y	U	Y	Y	Y	Y	High
[28]	Y	Y	Y	Y	Y	Y	Y	Y	Y	U	Y	Y	Y	Y	High
[29]	Y	Y	Y	Y	Y	Y	Y	Y	Y	Y	Y	Y	Y	Y	High
[30]	Y	Y	Y	Y	Y	Y	-	-	Y	Y	Y	Y	Y	Y	High
[31]	Y	Y	Y	Y	Y	Y	-	-	Y	Y	Y	Y	Y	Y	High

1. Did the study address a clearly focused issue? 2. Was the cohort recruited in an acceptable way? 3. Was the exposure accurately measured to minimize bias? 4. Was the outcome accurately measured to minimize bias? 5a. Have the authors identified all important confounding factors? 5b. Have they taken account of the confounding factors in the design and/or analysis? 6a. Was the follow-up of subjects complete enough? 6b. Was the follow-up of subjects long enough? 7. What are the results of this study? 8. How precise are the results? 9. Do you believe the results? 10. Can the results be applied to the local population? 11. Do the results of this study fit with other available evidence? 12. What are the implications of this study for practice?

**Table 3:** GRADE certainty of evidence for fluid biomarkers associated with cognitive or structural outcomes in athletes exposed to repetitive head impacts.

Study	Type	(n)	Classification Risk of Bias	Publication Bias	Inconsistency	Indirect Evidence	Imprecision	GRADE Certainty	Observations
[22]	Cohort	60	High	Low	N/A	Direct	Moderate	Moderate	Well-conducted study
[23]	Cohort	417	High	Low	N/A	Direct	Not critical	High	Adequate sample and follow-up
[24]	Cohort	472	High	Low	N/A	Direct	Not critical	High	Adequate sample and follow-up
[25]	Cross-sectional	47	High	Low	N/A	Direct	Serious	Very Low	Extremely small sample size
[26]	Cross-sectional	36	High	Low	N/A	Direct	Serious	Very Low	Extremely small sample size
[27]	Cohort	50	High	Low	N/A	Direct	Serious	Very Low	Extremely small sample size
[28]	Cohort	55	High	Low	N/A	Direct	Moderate	Moderate	Adequate sample and follow-up
[29]	Cohort	137	High	Low	N/A	Direct	Not critical	High	Adequate sample and follow-up
[30]	Cohort	235	High	Low	N/A	Direct	Not critical	High	Adequate sample and follow-up
[31]	Cross-sectional	158	High	Low	N/A	Direct	Not critical	High	Adequate sample and follow-up

Risk of bias: Based on the CASP checklist; “High” methodological quality corresponds to a low risk of bias (no downgrade), “Moderate” to a moderate risk (downgrade by one level), and “Low” to a high risk of bias (downgrade by two levels). Publication bias: Not formally assessed via funnel plots due to the small number of studies per outcome; therefore, not downgraded but discussed as a limitation. Inconsistency: assessed only when multiple outcomes from the same study or meta-analysis (N/A = not applicable). Indirectness: All included studies were rated as “Direct” as they specifically analyzed the correlation between fluid biomarkers and objective cognitive outcomes or structural imaging. Imprecision: Classified according to sample size and reporting of confidence intervals (CIs); “Not critical” (n ≥ 100 or CIs consistently reported, no downgrade), “Moderate” (n = 51–99, downgrade by one level), and “Serious” (n ≤ 50 or not reported, downgrade by one or two levels). GRADE certainty: Defined by integrating the five domains (risk of bias, inconsistency, indirectness, imprecision, and publication bias). Well-conducted observational studies started at “High” certainty and were downgraded based on the severity of identified limitations.

**Table 4:** Characterization of studies.

Study	Type of Study	Modality	General Characteristics	Exposure	Exposure Time
[22]	Cohort	American Football, Hockey, Cross-country, Ultimate frisbee (College athletes)	Contact (n = 20M/24F—20.4 ± 1.5 y); Non-contact (n = 9M/7F; 20.9 ± 1.1 y)	↑ RHI ↓ cognition	1 session of 24 and 48 h
[23]	Cohort	Boxing and MMA	Active MMA (n = 152M/17F; 29.6 ± 4.7 y), Control (n = 69M/10F; 30.8 ± 10.0); Active Boxing (n = 110M/7F; 30.4 ± 6.9 y); Retired Boxing (n = 50M/2F; 48.0 ± 10.2 y)	↑ RHI ↔ cognition	Active MMA (5.6 ± 4.2 y); Control (0); Active Boxing (5.9 ± 4.5 y); Retired Boxing (11.7 ± 5.3)
[24]	Cohort	Boxing e and MMA	Active Boxing (n = 130M/10F; 31 ± 8 y); Active MMA (n = 178M/33F; 30 ± 5 y); Retired Boxing (n = 65M/4F; 48.7 ± 9 y); Control (n = 39M/13F; 35.6 ± 12 y)	↑ RHI ↓ cognition	Active MMA (3.5 y); Control (0); Active Boxing (4.4 y); Retired Boxing (10.7)
[25]	Cross-sectional	Boxing, American football, Soccer, Hockey	ExPRO^+^ (n = 16M; 58.9 ± 11.4 y); ExPRO^−^ (n = 25M; 55.2 ± 6.9 y); Control (n = 6M; 54.0 ± 7.5 y)	↑ RHI ↔ cognition	ExPRO^+^ (10.1 ± 4.3); ExPRO^−^ (10.6 ± 3.4 y); Control (0)
[26]	Cross-sectional	Boxing, American Football, Soccer, Hockey, Karate, Wrestling	SRC (n = 13M/11F; 25 ± 5.1 y), Controls (n = 5M/7F; 28 ± 4.9 y)	↑ RHI ↔ cognition	Athletes (17.8 ± 5.0 y) and control (0)
[27]	Cohort	MMA	MMA (n = 48M/2F; 26.5 ± 5.8 y)	↑ RHI ↓ cognition	3 weeks post-fight
[28]	Cohort	Olympic boxers	Olympic boxers (n = 30M; 22 y); Controls (n = 25M; 22 y)	↑ RHI ↓ cognition	Chronic
[29]	Cohort	Former football players	SRC (n = 72M/9F; 22.8 y), Controls (n = 54M/2F; 24.6 y)	↑ RHI ↔ cognition	13.5 y SRC and control (14.0 y)
[30]	Cohort	American football	NBD^+^ (n = 104M; 58.2 ± 7.7 y); NBD^−^ (n = 76M; 58.2 ± 8.7 y); Control (n = 55M; 59.8 ± 8.3 y)	↑ RHI ↓ cognition	Chronic, NBD+ 16.3 y; NBD^−^ 15.2 y, control 0
[31]	Cross-sectional	Football players	Player (n = 120M; 57.5 ± 8.2 y); Control (n = 38M; 59.8 ± 8.9 y)	↑ RHI ↓ cognition	Chronic 16.3 y

TES = traumatic encephalopathy syndrome; HC = Healthy Control; MCI = Mild Cognitive Impairment; AD = Alzheimer Disease; SRC = sport-related concussion; RHI = repetitive head impacts; ExPRO^+^ = Former Professional Group; ExPRO^−^ = Former Professional Group; NBD^+^ = former American football players, with NBD diagnosis; NBD^−^ = former American football players without NBD diagnosis; MMA = mixed martial arts; SCD = sickle cell disease; PPCS = persistent post-concussive symptoms; n = sample size; y = years. Ages are expressed in years or age range. Exposure duration: chronic = prolonged exposure (career or post-retirement); acute/subacute = hours to weeks after the event. ↑ increased, ↓ decreased, ↔ no significant difference or no association.

**Table 5:** Clinical assessments, investigated biomarkers, and main findings.

Study	Cognitive Assessments	Biomarkers	Association with Cognition	Temporal Points
[22]	ImPACT (Reaction Time), PCS-R (self-reported of concussion)	Synaptic (Anti-GluA1, GluA1)	In contact-sport athletes, reaction time ↓ after the season (0.06 ± 0.19 vs. low contact 0.00 ± 0.04) and showed a low correlation (r = 0.37; *p* = 0.043) with the ↑ GluA1 autoantibodies, associated with ↓ reaction time only in this group.	Pre, post, and acute post-concussion phase, 24–48 h after injury, and return to sport
[23]	CNS Vital Signs (verbal memory, processing speed, attention span, executive function, psychomotor speed)	Axonal (NfL); neurodegenerative (Tau)	Active boxers showed + ↑ NF-L levels (21.55 pg/mL; *p* = 0.0001) vs. active MMA fighters (14.58 pg/mL), retired (15.12 pg/mL), and controls (11.27 pg/mL). Significant ↑ Tau over time only in active MMA fighters (*p* = 0.01). The ↑ tau was not associated with a change in cognitive performance. Basal + ↑ NF-L levels were associated with ↓ basal performance in the domains of psychomotor speed (r = −0.1219, *p* = 0.0203) and processing speed (r = −0.1097, *p* = 0.0378). However, no correlation was observed between basal tau level and performance on basal cognitive tests.	Annual monitoring for 5 years
[24]	CNS Vital Signs (verbal memory, processing speed, attention span, executive function, psychomotor speed)	Axonal (GFAP, NfL), neurodegenerative (p-tau231, NTA tau)	Basal GFAP ↑ in retired boxers vs. active MMA fighters (*p* = 0.019); NfL ↑ in active boxers vs. MMA (*p* = 0.047). ↑ GFAP was associated with cognitive ↓ (memory, processing speed, psychomotor speed, reaction time). In active fighters, ↑ GFAP correlated with ↓ psychomotor speed.	Baseline and annual, with an average follow-up of 2 to 4 years
[25]	RAVLT (immediate and delayed memory), Digit Span (working memory), PASAT (sustained attention and processing speed), Verbal Fluency (lexical production and executive function), Mood and behavior scales, PAI (anxiety, depression, mania, and aggression), WTAR (premorbid IQ estimate).	Axonal (NfL); neurodegenerative (p-tau181, Aβ1-42, and t-tau)	In the EXPRO^−^ group, 12% had abnormal pTau181, but without levels of MCI or AD. In the PRO^+^ group, 25% had abnormal pTau181, including cases in the MCI and AD ranges. Mean performance in executive functions, memory, and mood/behavior was normal in all groups, with no significant differences.	follow-up from 2011 to 2021
[26]	RBANS (attention, language, immediate memory, delayed memory, visuospatial/constructive), SCAT5 (self-reported of concussion)	Inflammatory (IL-2, IL-5, IL-7, IL-12/23p40, IL-15, IL-16, IL-17A, TNF-β, VEGF, IFN-γ, IL-1β, IL-4, IL-6, IL-8, IL-10, IL-13, TNF-α, Eotaxin, Eotaxin-3, IP-10, MCP-1, MCP-4, MDC, MIP-1α, MIP-1β, TARC.)	10 athletes showed impaired performance in cognitive tests. There was no strong correlation between most biomarkers and cognition or symptoms.	Acute (Post-injury)
[27]	Trail Making Test A and B (digital) (attention, processing speed, and executive function), Digit Span (working memory)	Axonal (UCHL1, MBP, NSE2, GFAP, S100B), Inflammatory (IL-6, VCAM-1, CCL2/MCP-1, CRP, ICAM-1), neurodegenerative (miRNA, BDNF)	Salivary and serum miRNAs predicted the probability of TBI and were associated with head impacts, cognitive measures, and balance. Serum proteins had much less utility.	Pre-fight (1 week before and 1 h before), post-fight (immediately, 15–30 min, 2–3 days, 1 week, and ≥3 weeks)
[28]	ROCF (memory), WAIS-R (memory), COWAT (verbal fluency), Digit Span (immediate memory), Listening Span Test (complex memory and attention), Trail Making Test A and B (attention, speed and cognitive flexibility), Reaction Time (simple and complex), Finger Tapping Test (motor speed).	Axonal (NfL, S100B, GFAP), neurodegenerative (tau total, BDNF, Aβ42)	Boxers vs. controls had more “know” responses in a visual memory task (*p* = 0.02); they performed better on working memory (*p* = 0.049). Boxers with prolonged ↑ NFL (after a rest period) showed deficits in Trail Making A (*p* = 0.041) and simple reaction time (*p* = 0.042).	1 to 6 days and 14 days after the fight
[29]	RPQ (Self-reported concussion symptoms), Cogstate Battery (psychomotor speed, attention, visual memory, working memory, reaction time, and accuracy)	Axonal (GFAP, NfL)	↑ GFAP and NfL levels are linked to greater clinical severity, loss of consciousness, and slower recovery, but the relationship with cognition is limited.	Applied at multiple points: 24 h, 1, 2, 4, 6, 8, 12, and 26 weeks post-injury.
[30]	Diagnosis of NBD, NINDS 2021 Criteria, Neurobehavioral dysregulation (explosiveness, emotional dyscontrol, affective lability, and impulsivity), Cognitive (verbal memory and executive functions, processing speed)	Axonal (NfL), Inflammatory (IL-1β, IL-6, IL-8, IL-10, CRP, TNF-α)	Former American football players with bipolar disorder had ↑ CSF IL-6, associated with ↑ emotional dyscontrol, affective lability, impulsivity, and total bipolar disorder scores. In older players, ↑ plasma NfL was associated with ↑ emotional dyscontrol and impulsivity, as well as ↓ executive function and processing speed.	We conducted baseline visits between September 2016 and February 2020
[31]	NINDS criteria, Neurobehavioral dysregulation (explosiveness, emotional dyscontrol, affective lability, and impulsivity), Cognition (Learning, verbal memory, executive function, psychomotor speed, visual memory), Number span (Verbal fluency), Mood scales, BDI-II, and BAI.	Neurodegenerative (NE, DHPG, DA, L-DOPA, DOPAC)	Former players showed ↓ NE, L-DOPA, and DOPAC vs. controls. In the COL subgroup, ↑ NE and ↑ L-DOPA were associated with worse executive function/psychomotor speed, higher impulsivity, and emotional dyscontrol.	We conducted baseline visits between September 2016 and February 2020

Aβ, amyloid beta; ANT, Attention Network Test; AUC, area under the curve; BAI, Beck Anxiety Inventory; BDI-II, Beck Depression Inventory—Second Edition; BESS, Balance Error Scoring System; CDR-SB, Clinical Dementia Rating—Sum of Boxes; CN, cognitively normal; CNS, Central Nervous System; COWAT, Controlled Oral Word Association Test; CRP, C-reactive protein; CSF, cerebrospinal fluid; CTE, chronic traumatic encephalopathy; DA, dopamine; DEM, dementia; DHPG, 3,4-dihydroxyphenylglycol; DMN, default mode network; DOPAC, 3,4-dihydroxyphenylacetic acid; fMRI, functional magnetic resonance imaging; FPN, frontoparietal network; GFAP, glial fibrillary acidic protein; HAM-D, Hamilton Depression Rating Scale; IL, interleukin; IL-1RA, interleukin-1 receptor antagonist; ImPACT, Immediate Post-Concussion Assessment and Cognitive Testing; L-DOPA, levodopa; MBP, myelin basic protein; MCI, mild cognitive impairment; miRNA, microRNA; MMSE, Mini-Mental State Examination; NBD, neurobehavioral dysfunction; NE, norepinephrine; NfL, neurofilament light chain; NGS, next-generation sequencing; NK, natural killer; NSE, neuron-specific enolase; NTA, N-terminal tau; PCS, post-concussion symptoms; PCS-R, Post-Concussion Symptom Scale—Revised; PET, positron emission tomography; PPCS, persistent post-concussion symptoms; P-tau, phosphorylated tau; RAVLT, Rey Auditory Verbal Learning Test; RBANS, Repeatable Battery for the Assessment of Neuropsychological Status; RNA-seq, RNA sequencing; ROI, region of interest; SAC, Standardized Assessment of Concussion; SCAT, Sport Concussion Assessment Tool; SCAT5, Sport Concussion Assessment Tool—5th Edition; SMC, subjective memory complaint; SN, salience network; TARC, thymus and activation-regulated chemokine; TES, traumatic encephalopathy syndrome; TNF, tumor necrosis factor; UCH-L1, ubiquitin C-terminal hydrolase L1; UCSF, University of California, San Francisco; VAT, Verbal Association Test; VEGF, vascular endothelial growth factor. ↑ increased, ↓ decreased.

## Data Availability

Not applicable. No datasets were generated or analyzed during the current study. The literature search strategy used to identify relevant studies is provided as Supplementary Materials.

## Supplementary Materials

The following supporting information can be downloaded at https://www.mdpi.com/article/10.3390/sports14070272/s1.
