# Supplementary material for: Biological Markers of Cognitive Impairments in Combat and Contact-Sport Athletes: A Systematic Review
Source: Sports (Basel). 2026 Jul 1;14(7):272. doi: 10.3390/sports14070272 (PMC13417074; doi:10.3390/sports14070272)
Supplement: Supplementary file 1 [file sports-14-00272-s001.zip › sports-4248344-supplementary.pdf]

Search: Biological Markers of Cognitive Impairments in Contact Sports Athletes: A Systematic Review

APA PsycNet

Search date: April 11, 2026

Fields searched: Title, abstract, keywords

Full strategy: biomarker* OR "biological markers" OR "blood biomarkers" AND "cognitive impairment" OR "cognitive decline" OR "cognitive dysfunction" OR "neurocognitive impairment" OR "executive dysfunction" AND athletes OR "contact sports" OR "combat sports"

Limits/filters: None

Number of records: 1,083

PubMed

Search date: April 11, 2026

Fields searched: Title, abstract, MeSH terms

Full strategy: (biomarker* OR "biological markers" OR "blood biomarkers") AND ("cognitive impairment" OR "cognitive decline" OR "cognitive dysfunction" OR "neurocognitive impairment" OR "executive dysfunction") AND (athletes OR "contact sports" OR "combat sports")

Limits/filters: None

Number of records: 4,894

VHL – Virtual Health Library

Search date: April 11, 2026

Fields searched: Title, abstract, subject (DeCS)

Full strategy: biomarkers OR biomarkers OR "serological biomarkers" OR "cerebrospinal fluid biomarkers" AND "cognitive impairment" OR "cognitive impairment" AND "combat sports" OR "contact sports"

Limits/filters: None

Number of records: 494

Google Scholar

Search date: April 11, 2026

Fields searched: Title, abstract, subject (DeCS)

Full strategy: biomarkers OR biomarkers OR "serological biomarkers" OR "cerebrospinal fluid biomarkers" AND "cognitive impairment" OR "cognitive impairment" AND "combat sports" OR "contact sports"

Limits/filters: None

Number of records: 2,920

CAPES Journal Portal

Search date: April 11, 2026

Fields searched: Title, abstract, subject (DeCS)

Full strategy: "biomarkers" AND "cerebrospinal fluid" AND "executive function" AND "contact sports" OR "combat sports"

Limits/filters: None

Number of records: 1,083
